# A Guide through the Dental Dimethacrylate Polymer Network Structural Characterization and Interpretation of Physico-Mechanical Properties

**DOI:** 10.3390/ma12244057

**Published:** 2019-12-05

**Authors:** Izabela Maria Barszczewska-Rybarek

**Affiliations:** Department of Physical Chemistry and Technology of Polymers, Silesian University of Technology, Strzody 9, 44-100 Gliwice, Poland; Izabela.Barszczewska-Rybarek@polsl.pl

**Keywords:** dental materials, dimethacrylates, polymer networks, structure, morphology, degree of conversion, crosslink density, physical crosslinking, hydrogen bonds, mechanical properties, water sorption

## Abstract

Material characterization by the determination of relationships between structure and properties at different scales is essential for contemporary material engineering. This review article provides a summary of such studies on dimethacrylate polymer networks. These polymers serve as photocuring organic matrices in the composite dental restorative materials. The polymer network structure was discussed from the perspective of the following three aspects: the chemical structure, molecular structure (characterized by the degree of conversion and crosslink density (chemical as well as physical)), and supramolecular structure (characterized by the microgel agglomerate dimensions). Instrumental techniques and methodologies currently used for the determination of particular structural parameters were summarized. The influence of those parameters as well as the role of hydrogen bonding on basic mechanical properties of dimethacrylate polymer networks were finally demonstrated. Mechanical strength, modulus of elasticity, hardness, and impact resistance were discussed. The issue of the relationship between chemical structure and water sorption was also addressed.

## 1. Introduction

Poly(dimethacrylate)s are highly crosslinked polymer networks, irreplaceable in applications requiring fast polymerization processes, such as light-curing dental materials [1,2,3,4,5]. A structural diversity, low manufacturing costs, excellent aesthetics, and easy handling have determined the utilization of poly(dimethacrylate)s mainly as matrices in dental restorative composites [6,7,8,9] and provisional restoration materials [10].

Dental composites usually consist of a dimethacrylate resin, inorganic filler (usually silica particles of various sizes—nanofillers, microfillers, and macrofillers or the combination of the latter two, which is called “hybrids” [11]), polymerization initiating system, silane coupling agent, which binds organic matrix with a filler, and pigments [6,7,8,9]. The majority of dental materials are single-component photocured systems. Photocuring is usually carried out with the aid of camphorquinone initiator and a tertiary amine reducing agent (usually, *N*,*N*-dimethylaminoethyl methacrylate) [12], under visible light irradiation within the range of high-intensity blue light (470–490 nm) [9]. There are also chemically cured, two-component systems. They contain a peroxide initiator (usually, benzoyl peroxide) and an amine accelerator (usually, *N*,*N*-dimethyl-p-toluidine) [9,12].

**Scheme 1:**
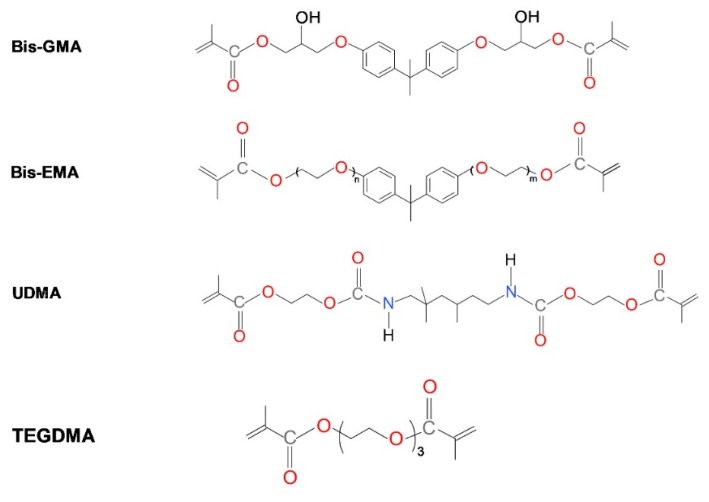
The chemical structure of popular dental dimethacrylate monomers.

The most important dental dimethacrylate monomers include 2,2-bis-[4-(2-hydroxy-3-methacryloxypropoxy)phenyl]propane (Bis-GMA, bisphenol A glycerolate dimethacrylate), bisphenol A ethoxylate dimethacrylate (Bis-EMA), 1,6-bis-(methacryloyloxy-2-ethoxycarbonylamino)-2,4,4-trimethylhexane, which is called the urethane-dimethacrylate monomer (UDMA), and triethylene glycol dimethacrylate (TEGDMA) (Scheme 1) [6,7,8,9]. The dimethacrylate monomers, due to their easy preparation and sufficient working time after light exposure, give an edge to design soft actuable (controlled movable carrier) for oral drug delivery not only for the oropharmacological products but other soft tissues in vitro and in vivo, as mentioned by Singh et al. [13]. 

Dimethacrylates are usually used in mixtures of different ratios and then copolymerized [9,14]. There are following exemplary combinations of dimethacrylates used in commercial composites: Bis-GMA and TEGMA (Clearfil ST^®^ (Kuraray), Grandio^®^ (VOCO), Filtek Z100^®^ (3M ESPE)), Bis-GMA, UDMA and TEGDMA (FSB^®^ (3M ESPE), Tetric Ceram^®^ (Ivoclar Vivadent)), Bis-GMA, UDMA and Bis-EMA (Filtek Z250^®^ (3M ESPE)) [14]. 

Bis-GMA, patented by Bowen in 1962 [15], was the first dental dimethacrylate resin [6,7,8,9]. Its large molecular weight and low concentration of double bonds (Table 1) provide low volatility, low polymerization shrinkage, rapid curing, and stiff, durable products of curing [16]. The extremely high viscosity of Bis-GMA limits the degree of conversion and decreases the possibility of filler incorporation. The viscosity of Bis-GMA can be lowered by admixing low molecular weight dimethacrylates. Oligoethylene glycol dimethacrylates may be used for this purpose, of which TEGDMA is the most popular. The lower the viscosity of the mixture, the higher the degree of conversion and the more filler can be incorporated [16]. However, the addition of TEGDMA causes an increase in polymerization shrinkage [16]. In response to Bis-GMA flaws, Bis-EMA and UDMA monomers were developed. These monomers have similar molecular weights to Bis-GMA but are less viscous. Bis-EMA, having no hydroxyl groups is less viscous than UDMA. The mixture of Bis-GMA and Bis-EMA can be used without a reactive diluent, e.g., Renamel^®^ (Cosmedent) [14]. Spectrum TPH^®^ (Dentsply) also does not contain TEGDMA, as it is composed of Bis-GMA, UDMA, and Bis-EMA [14]. The higher viscosity of the UDMA monomer results from the formation of intermolecular hydrogen bonds between urethane species. Due to the good mechanical properties of the UDMA homopolymer, it is the only dimethacrylate that can be used alone in commercial composites, such as Lava Ultimate^®^ (3M ESP) [17]. It can also be combined with Bis-GMA, acting as a viscosity reducer, e.g., Tetric EvoCream^®^ (Vivadent Ivoclar) [14]. 

The precise explanation and understanding of the physicomechanical behavior of dimethacrylate polymer networks is a difficult task. Years of research have shown that their properties result from the interaction of the following structural factors: the monomer chemical structure, polymer network molecular structure (crosslink density, which relates to the chemical crosslink density, physical crosslink density, and degree of conversion), as well as morphology.

**Figure 1:**
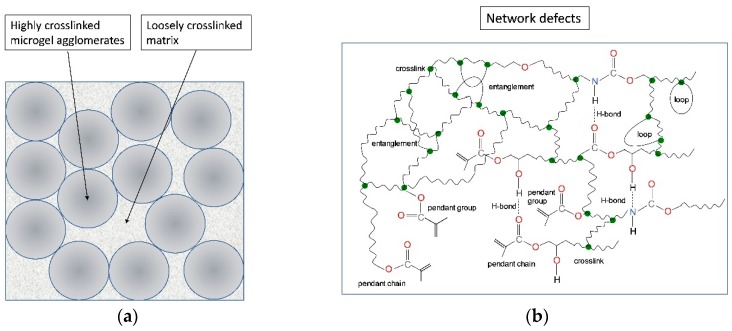
The structural heterogeneity of dimethactylate polymer networks: (**a**) schematic representation of the morphology; (**b**) defects in the polymer network microstructure.

As far back as the 2000s, the kinetics of dimethacrylate polymerization was precisely described. The theory of the ideal polymer network was developed at that time [20,21,22]. The real network was characterized by specifying its defects, such as pendant groups and chains, loops, entanglements, and sol fraction (Figure 1) [4,9,21,23]. Attention was paid to stochastic and spatial correlations as well as diffusion control of the reaction [1,2,3,4,9,20]. These studies have shown, that the radical polymerization of dimethacrylates is a complex process and involves a series of phenomena, such as auto acceleration (gel effect, occurring when the degree of conversion is only 1–2%), auto deceleration (observed in the further stages of the reaction), the reaction diffusion (the mechanism controlling termination), incomplete conversion of functional groups, the formation of microgel agglomerates (clusters of highly crosslinked polymer of a high degree of cyclization, suspended in the less cross-linked matrix). In effect, such polymer networks are spatially heterogeneous and consist of clusters varying in crosslink density, in which the degree of double bond conversion (*DC*) is never full (Figure 1) [1,2,3,4,20,21,22,23,24,25]. 

With scientific development, the need to clarify the influence of the network’s structural features on the material properties has become apparent. The issue of a comprehensive analysis of the structural heterogeneity of dimethacrylate polymer networks, based on a quantitative analysis of each structural aspect arose. A supramolecular structure required quantitative characterization. At the same time, in the literature, it had been suggested, that the presence of microgel agglomerates may adversely affect mechanical properties of poly(dimethacrylate)s, primarily the impact resistance [21,24,25,26,27,28]. In recent years, with advances in characterization techniques, substantial efforts have been made to precisely describe the polymer network spatial heterogeneity, especially the morphology [29,30,31,32,33,34,35,36,37,38,39,40,41,42,43,44,45]. All of this has resulted in many studies on structure-property relationships. Having expertise in this field is in demand for understanding properties of currently used dimethacrylate polymers as well as for designing new dimethacrylate monomers and their compositions in order to achieve the best possible material efficiency. 

The purpose of this paper is to review the current state of knowledge on the relationships between structure and properties in the crosslinked matrix of dimethacrylate based dental materials. As their structural characterization is a complex issue, an explanation of the influence of each structural aspect on physicomechanical properties was consistently undertaken. In addition, the article describes major techniques that are currently used to quantitatively analyze the structure of poly(dimethacrylate)s, with reference to the recent developments in this area. Issues presented were based on studies of typical dental dimethacrylate polymer networks as well as on other dimethacrylate polymers, described only in the literature. The analysis of the latter provided support for conclusions on the structure-property relationships, which may be especially useful when defining future trends in the design of new dental dimethacrylate systems. 

## 2. The Chemical Structure

The chemical structure of the dimethacrylate monomer might be recognized as a crucial factor in determining the physicochemical and mechanical performance of the corresponding polymer network. The mechanisms of its action can be divided into direct and indirect effects. 

The direct action relates to the influence of chemical groups, constituting the monomer structure, on polymer properties. It also determines the theoretical crosslink density and physical crosslinking.

The indirect action relates to the influence of the monomer chemical structure on the degree of conversion, real crosslink density as well as the supramolecular organization in the corresponding polymer and their consequences in polymer properties. 

Speaking of the direct action, it might be said that the dimethacrylate chemical structure determines the overall elasticity of the crosslinked material [22]. Composite dental materials require highly crosslinked polymers that offer high stiffness, hardness, and durability in order to be able to withstand the stresses exerted by orthodontic mechanics and variations in the oral environment [46]. 

Firstly, the dimethacrylate molecule length determines the theoretical crosslink density of a corresponding polymer network. The shorter the distance between double bonds, the higher the crosslink density. An increase in crosslink density causes a decrease in the possibility of chain reorganization [5,18,31,32,35,47]. Consequently, an increase in modulus is observed [19,23,48]. Physical crosslinks, such as hydrogen bonds, reduce the rotational motion too, causing a stiffening of the network structure [3,49,50,51,52,53]. 

Secondly, the dimethacrylate monomer molecular elasticity determines the general network elasticity [21,22]. Aliphatic hydrocarbon and oligoether chains give rise to an increase in polymer elasticity, whereas cycloaliphatic and aromatic units cause an increase in polymer stiffness [31,32,48,54,55,56]. 

**Scheme 2:**
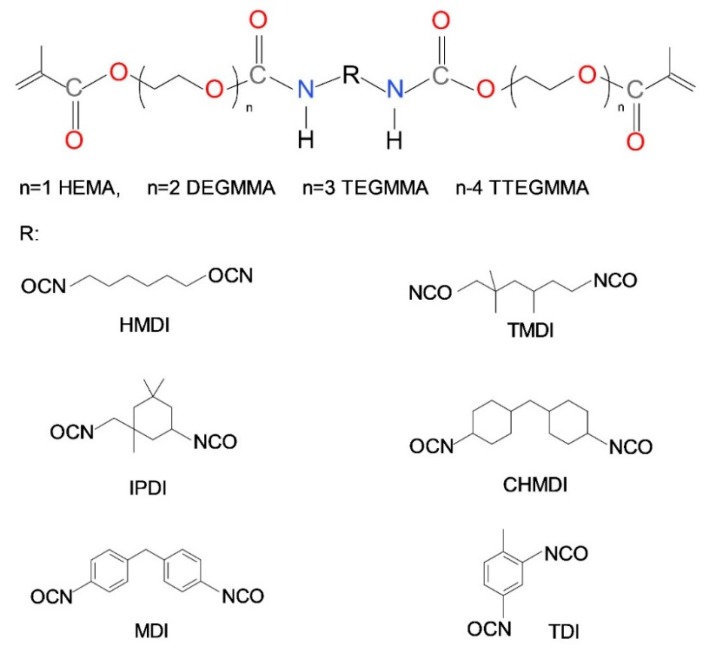
The chemical structure of a series of urethane-dimethacrylate monomers.

Water sorption (*WS*) can be also regarded as the chemical structure-dependent physical property of dimethacrylate polymer networks. A certain swelling capacity can be beneficial in dental applications. It was found that polymerization shrinkage deformation can be compensated by hygroscopic expansion. However, the scale of this expansion in dental materials cannot exceed the polymerization shrinkage [57]. For these reasons, the *WS* of 50 μg/mm^3^ was established as an upper limit for dental restorative materials [58]. Like the elastic behavior, water sorption depends on the intrinsic action of the theoretical crosslink density, resulting from both covalent and non-covalent crosslinks, as well as monomer chemical structure, which determines chain elasticity and hydrophilicity. The longer the chains between crosslinks, the lower the crosslink density, the higher the water sorption [48,52,53]. Especially, highly elastic hydrocarbon or oligoether chains give rise to the accommodation of higher water quantity. This effect was shown by several works on *WS* of dimethacrylate polymers, consisting of oligooxyethylene chains. Park et al. studied a series of Bis-GMA copolymers with mono-, di- and triethyleneglycol dimethacrylates [52]. Ogliari et al. tested the influence of the degree of Bis-EMA ethoxylation on *WS* [54]. Barszczewska-Rybarek studied *WS* of homopolymers of six homologous series of urethane-dimethacrylates (Scheme 2) [48]. An increase in the ethylene oxide number in a chain always resulted in an increase of water sorption. Additionally, it was found that if the number of oxyethylene units was greater than three, the *WS* significantly exceeded the limit of 50 μg/mm^3^.

The study on urethane-dimethacrylate polymer networks additionally provided results for the influence of the diisocyanate core on *WS*. *WS* increased according to the following order of diisocyanates: Symmetrical cycloaliphatic < symmetrical aromatic < asymmetrical aromatic < substituted aliphatic chain < asymmetrical cycloaliphatic < linear aliphatic chain [48]. This order shows that *WS* depends on the combination of the diisocyanate chemical character and its symmetry. Generally, fully aliphatic polymers had higher *WS* than those, having aromatic and alicyclic structures. If compared to the influence of only aliphatic diisocyanates on *WS*: The linear HMDI and substituted with methylene groups TMDI, the presence of the latter resulted in higher *WS*. 

**Scheme 3:**
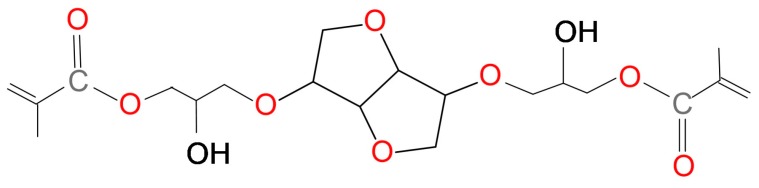
The chemical structure of IS-DMA.

The influence of cycloaliphatic ring on *WS* was not defined clearly enough. Both aromatic structures (regardless of the molecular symmetry) and symmetrical cycloaliphatic CHMDI caused a decrease in *WS*. The presence of asymmetrical cycloaliphatic IPDI resulted in a significant *WS* increase. This result can be confirmed by findings of Łukaszczyk et al. The comparative analysis of the influence of Bis-GMA and its isosorbide analogue (IS-DMA) (Scheme 3) on *WS* of their homopolymers and copolymers with TEGDMA 40 wt% was performed in their work [59]. 

It was found that *WS* of IS-DMA homo- and copolymer (respectively, 173 and 100 μg/mm^3^) was much greater than *WS* of their Bis-GMA analogues (respectively, 11 and 15 μg/mm^3^). IS-DMA differs from Bis-GMA by the diol core, deriving from isosorbide (a bicyclic diol, consisting of two fused tetrahydrofuran rings). Polymers of both types were characterized by a similar molecular weight, degree of conversion and mechanical properties [59]. The observed differences in *WS* were likely due to the reduced strength of intermolecular interactions. It can be explained by the large size of the space the isosorbide occupies and its irregular shape. As a result, larger empty space might be present within polymer network clusters. From these reasons water can easily penetrate through the IS-DMA matrix and accumulate, being caught by hydroxyl, ester and ether groups.

The influence of the presence of hydrogen bond proton donor groups on water sorption exhibits another interesting issue. Obviously, hydroxyl and urethane groups promote water swelling as they can form strong hydrogen bonds involving proton donor as well as proton acceptor atoms [60,61]. That would explain the higher *WS* of the Bis-GMA homopolymer, having two pendant hydroxyl groups, than the OH-free Bis-EMA homopolymer [53]. On the other hand, the *WS* of highly crosslinked TEGDMA homopolymer is significantly higher (Table 2). It can be attributed to the lack of proton donors, resulting in an inability to form strong hydrogen bonds and the presence of highly elastic oligoether chains in the TEGDMA molecule [50,53,58]. It leads to the general conclusion that physical crosslinking, resulting from hydrogen bonding, can tighten a polymer network structure and, to some extent, limit water swelling. 

## 3. The Chemical Crosslink Density

As mentioned above, the theoretical crosslink density (*q_theor_*) of dimethacrylate polymer networks is of great importance for describing the general tightness of the polymer network. 

A dimethacrylate polymer network structure differs from the structure of a typical crosslinked polymer obtained by the polymerization of difunctional methacrylate with a certain amount of a tetrafunctional dimethacrylate crosslinker [62,63]. In that latter type of a crosslinked structure, a dimethacrylate molecular weight can be neglected as its body is treated as a volumeless tetrafunctional crosslink, connecting polymethacrylate chains. Some works on dimethacrylate polymer networks lead to the conclusion that, in structural considerations, the repeating unit can be recognized as a primary chain ending at both ends with two volumeless trifunctional crosslinks –CH_2_C(CH_3_)– [64,65]. It justifies making an approximation that the dimethacrylate monomer molecular weight (*MW*) corresponds to the network parameter (*M_c_*)–the average molecular weight between cross-links [21]. In this way, the concentration of double bonds (*X_DB_*) can be used as a measure of the theoretical crosslink density. The method of calculating *q_theor_* from *MW* in relation to *X_DB_* was used in several works [18,31,35,66,67]. Dimethacrylates with higher *MW* have lower *X_DB_* and therefore they are expected to form networks of lower *q_theor_*.

The theoretical crosslink density never corresponds to the real crosslink density (*q*) [21]. The crosslink density in real systems results from *q_theor_* and other factors, such as the incomplete conversion and presence of loops [1,2,3,4,5,23,24,25]. As the degree of conversion (*DC*) is never full in dimethacrylate polymer networks, the real crosslink density is therefore always lower than its theoretically calculated value. Loops, although they do not cause a decrease in the *DC* (both chain ends are attached to the same junction point [21]), they do cause a decrease in *q* (Figure 1). 

Several propositions of calculating the real crosslink density (*q*) are presented in the literature.

In the work of Barszczewska-Rybarek et al., *q* of a series of urethane-dimethacrylate polymer networks (Scheme 2) was calculated from the degree of conversion (*DC*). The following formula was developed [68]:

(1) $$q=\frac{2DC-1}{DC}$$

In the work of Sideridou et al. this equation was further developed by using the mass of unreacted monomer [53]:

(2) $$q=\frac{2DCm_{1}-m_{3}}{DCm_{1}}$$

where *m*_1_ is the mass of the dried discs obtained through polymerization, *m*_3_ is the mass of the dried discs after water extraction.

Another approach was presented in the work of Barszczewska-Rybarek [35]. The concentration of double bonds in the UDMA monomer (*X_DB_*) was reduced by the fraction of unreacted double bonds and the obtained value was treated as a measure of the real crosslink density (*q*). *q* was calculated using the following equation:

(3) $$q=X_{DB}\times DC$$

The crosslink density may also be calculated from the network parameter (*M_c_*). *M_c_* is usually determined by utilizing dynamic mechanical analysis [29,65,68,69,70,71,72] and in swelling studies [73], following well known Flory–Rhener equation [74]. 

The literature provides several equations, which relates *M_c_* to *q*. The best known and most commonly used is the following equation [75]:

(4) $$M_{c}=\frac{MW}{q}$$

where *MW* is a monomer molecular weight.

However, when Equation (4) is applied to highly crosslinked dimethacrylate polymer networks it may give inadequate results [22,29,65,68]. In order to better characterize the structure of poly(dimethacrylate)s the modifications of Equation (4) were developed. Assuming a dimethacrylate molecule to form one tetrafunctional crosslink, the following equation was constructed [65]:

(5) $$M_{c\left(f=4\right)}=\frac{MW}{q}-\frac{MW}{2}$$

Assuming a dimethacrylate molecule to form two trifunctional crosslinks the following equation was constructed [65]:

(6) $$M_{c\left(f=3\right)}=\frac{2MW}{3q}-\frac{MW}{3}$$

To summarize, it can be said that the network is tighter as *q* increases and *M_c_* decreases. If *M_c_* is greater than *MW* and *DC* is higher than 50%, this procedure can be used for describing the polymer network tightness, otherwise, the value of *q* does not have a physical meaning [29,68].

## 4. The Physical Crosslink Density

**Figure 2:**
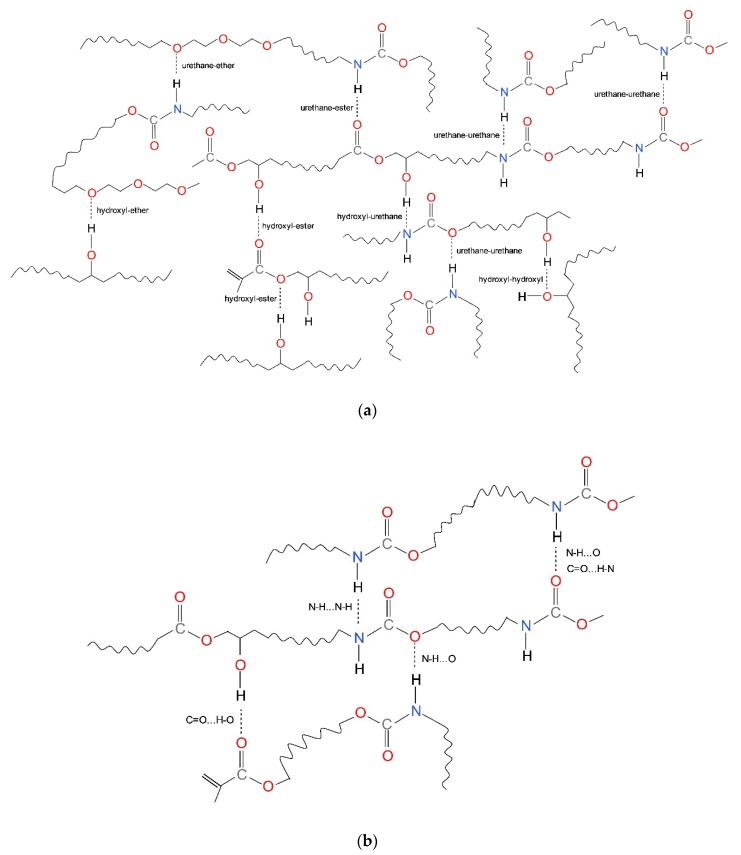
Hydrogen bonds in dental dimethactylate polymer networks (based on [76]): (**a**) various possibilities for hydrogen bonding; (**b**) hydrogen bonds narrowed to examples presented in Table 3.

The physical crosslinking in dental dimethacrylate polymer networks results from hydrogen bonding. A wide range of hydrogen bond options in dental poly(dimethacrylate)s are shown in Figure 2. 

Homopolymers and copolymers consisting of Bis-GMA and/or UDMA are therefore crosslinked both chemically and physically. TEGDMA can be involved in physical crosslinking by the acceptor oxygen atom of the ester and ether groups. However, this is only possible if TEGDMA is copolymerized with another dimethacrylate, which can provide a proton donor. This means that the TEGDMA homopolymer cannot be physically crosslinked.

Hydrogen bonding determines the dimethacrylate monomer viscosity. The higher the dissociation energies of hydrogen bonds, which can be formed in the system, the stronger the H-bonds and the higher the monomer viscosity [3,31,45,50,77]. Small molecule monomers, such as TEGDMA, that lacks an H-bond proton donor present very low viscosities (0.05 Pa∙s). On the other hand, Bis-GMA exhibits a dramatically high viscosity (from 700 to more than 1300 Pa∙s [6,18,19,31]. This is the result of the formation of strong intermolecular hydrogen bonds involving hydroxyl donors. 

The influence of hydrogen bonding on a monomer viscosity can be represented by the comparison of Bis-GMA viscosity with its ethoxylated analogue. Bis-GMA is about a thousand times more viscous than Bis-EMA (Table 1) [18,19]. 

Assuming the analogy in the monomer and polymer chemical structure, this dependence was adopted for the characterization of the strength of physical interactions in a polymer. The overall strength of intermolecular interaction was considered to have followed a trend like viscosity, i.e., the higher the monomer viscosity, the higher the overall strength of physical interactions in a polymer network [3,31,45,66,77,78].

Another approach to quantifying physical crosslinking was based on the analogy between the intermolecular interactions in the solutions of urethane-dimethacrylate monomers (Scheme 2) and in the corresponding polymers. The concentration dependence on 1H-NMR chemical shifts of the NH urethane protons was employed for this purpose [65]. The association constants were then calculated using the Benesi–Hildebrand equation [79]. Their values were discussed from the perspective of the monomer wing length and the core symmetry (only monomers with aromatic MDI and TDI cores were tested). The longer oligooxyethylene chain–the lower the association constant. The presence of asymmetrical TDI resulted in lower association constants if compared to the symmetrical MDI.

Pfeifer et al. [5], as well as Lemon et al. [50], estimated the strength of hydrogen bonds by utilizing FTIR spectroscopy in the studies on poly(dimethacrylate)s. The wavenumber and intensity of the -OH absorption peak at approximately 3500 cm^−1^ were monitored. They assumed that, the longer the peak maxima wavelength and the higher the -OH absorbance, the higher the strength of hydrogen bonds. Yilgör et al. provided FTIR data about changes in the ν(N–H) and ν(C=O) band location resulting from hydrogen bonding [80] and Antonucci et al. provided data for the ν(C=O) band location [81]. Those results confirm the correctness of the methodology used in the Pfeifer and Lemon works.

The literature also provides the physical crosslink density calculation procedure, which is analogue to the procedure being utilized to determine the theoretical chemical crosslink density from the concentration of double bonds in a monomer and its molecular weight. The physical crosslink density was calculated from the number of urethane bonds in a series of urethane-dimethacrylate polymer networks (Scheme 2) [35].

## 5. The Degree of Conversion

The degree of double bond conversion (*DC*) is the most evident parameter, defining the dimethacrylate polymer network structure. This is also the most often used parameter when the structure-property relationships are being investigated. 

The *DC* in poly(dimethacrylate)s is never full [1,2,3,4,5,6,7,8,9,18,19,23,24,25]. Conventional composite dental materials usually reach a *DC* of about 50–75% [6,8,9,47,82,83,84]. The limiting conversion in homopolymer networks of spacious dimethacrylates, such as Bis-GMA, can be even less than 50 % [18,19,31,47]. Polymer networks, characterized by the *DC* lower than 50% are unacceptable in practical applications due to the presence of a sol fraction [1,3,5,68,82,85]. Some authors suggest a *DC* of 55% as a minimum for clinical success in dentistry [82]. Insufficient curing causes a radical distortion of physicochemical and mechanical behavior as well as decreases material biocompatibility [19,25,85,86]. The leaching of a soluble fraction can cause tissue irritation and have a more serious effect on the organism [87,88]. For these reasons, monomers are usually copolymerized, which provides a satisfactory level of curing and properties.

The *DC*, in general, depends on the monomer chemical structure [18,19,31,54,77,78,83,89,90,91]. However, several polymerization parameters, such as: initiation technique [92], curing time [5], sample thickness [84,93], initiator system and its concentration [31,94,95,96,97,98], filler content [99], irradiation time [83], and source [83,97] also play a significant role.

### 5.1. Methods of the DC Determination

Several instrumental methods are available to allow the *DC* determination in dimethacrylate polymer networks. The infrared spectroscopy (FTIR, ATR-FTIR, NIR), Raman spectroscopy (RS), differential scanning calorimetry (DSC) and solid-state nuclear magnetic resonance (ssNMR) are particularly readily used.

#### 5.1.1. Fourier Transformed Infrared (FTIR) Spectroscopy

FTIR is the most popular method for the determination of *DC* in poly(dimethacrylate)s. Polymer samples to be analyzed by FTIR can be prepared in various forms. Usually, they are powdered and then mixed, in small amounts, with a dry potassium bromide (KBr) powder [18,29,45,59,90,92,98,99,100,101,102]. In recent works attenuated total reflection (ATR) sampling technique was effectively used for the *DC* determination [85,91,103,104,105,106,107,108]. The main advantage of the ATR-FTIR is simplicity in sample processing. Samples are examined directly in the solid or liquid state without further preparation [109,110]. In the case of the real-time experiments, a monomer drop is sandwiched between NaCl or KBr plates and then polymerized directly in a measuring chamber of the apparatus [5,29,31,54,91,108].

To determine the *DC* in dimethacrylate polymer networks, the near (NIR, from 4000 to approximately 14,000 cm^−1^) and mid (MIR, from 400 to 4000 cm^−1^) spectral infrared regions can be used [83,101]. However, measurements in the MIR region remains fundamental. As Fourier transform spectrometers typically work in the mid-infrared, this technique is abbreviated as FT-IR [111].

Regardless of the spectral region to be used, the determination of the *DC* in dimethacrylate systems is based on the monitoring of the absorption intensity of double bond vibrations, which decreases due to polymerization. The decreasing absorption intensity of the C=C bonds can be monitored with the use of several bands, present on the spectrum, which refers to the C=C bond various deformations. Their location and intensity depend on the spectrometer working infrared region (NIR and MIR).

The dimethacrylate group provides bands in the FTIR spectra at 1637 cm^−1^, referring to the C=C stretching vibrations [18,29,54,85,90,91,92,98,99,100,103,104,105,106,107,108,109,110,111,112,113,114,115,116,117,118,119,120], 948 cm^−1^–referring to the =CH_2_ wagging and 816 cm^−1^–referring to the =CH_2_ twisting [112]. Since the peak at 1637 cm^−1^ is stronger [112], it is most commonly used.

In the dimethacrylate NIR spectra, absorption of =C–H bond is located at about 4743 cm^−1^, while the first overtone is at 6165 cm^−1^. Because the baseline drops sharply in the 4743 cm^−1^ region, peak area measurements do not provide reliable results. Conversely, the region at 6165 cm^−1^, is very stable and there is no ambiguity in baseline construction [95]. Therefore, the methacrylate double bond conversion is determined by measuring the overtone band at 6165 cm^−1^ [5,19,31,66,67,81,83,93,95,101,109,121,122,123].

Since the Beer–Lambert law requires equal sample thickness, the direct ratioing of the C=C absorbance intensity in the polymer and monomer samples is not applicable [101]. For this reason, the relative band ratio method is usually used for the *DC* determination in poly(dimethacrylate)s [101]. This is, of course, approximation, because, due to the polymerization, the refractive index and the intermolecular interactions in the monomer and polymer are altered [124]. In the internal standard method, the intensity of the double bond vibrations is related to the intensity of the internal standard. This is a band, referring to the bond, whose absorptivity in the monomer and in the polymer is unaffected by a polymerization [101]. The *DC* is then calculated according to the following general formula [18,29,83,85,90,91,92,98,99,100,101,103,104,107,112,114,115,116,117,118,119,125]:

(7) DC(%)=[1−(AC=CAIS)pol(AC=CAIS)mon]×100

where *A_C=C_* is the absorbance of the C=C double bonds, *A_IS_* is the absorbance of the internal standard, *pol* is polymer, *mon* is monomer.

One of the most valuable series of bands utilized as an internal standard derive from in-plane skeletal stretching vibrations of the C=C aromatic ring, occurring in the range of 1620 to 1565 cm^−1^ [101,112]. The band at 1608 cm^−1^ is mostly used for this purpose [18,83,85,90,91,92,98,99,100,103,107,115,116,117]. The second band, at 1582 cm^−1^ can also be used, however, it is usually weaker [82,103]. An aromatic band is present at 4623 cm^−1^ too. It is not recommended to be used as an internal standard, because depending on the dimethacrylate chemical structure, it can undergo a slight variation in its intensity, introducing an error in the *DC* determination [121].

If the monomer contains no aromatic structures, the band from the C=O stretching vibrations can be used as an internal standard. It is located at around 1715–1720 cm^−1^ [18,29,90,91,101,104,112,114,118,119,125]. It should be noted, that this method produces underestimated *DC* values [90,91]. It was demonstrated in the studies on a series of Bis-GMA/TEGDMA and Bis-GMA/Bis-EMA copolymers. The average difference between the *DC* measured with the carbonyl and aromatic internal standards was, respectively of 23% and 17% [91]. Variations were also observed in the *DC* of urethane-dimethacrylate homopolymers (Scheme 2, TDI and MDI series) [90]. The difference between *DC* values obtained with the use of the carbonyl and aromatic internal standards depended on the monomer molecule length and decreased from 51% to 0% as the monomer molecule length increased. The reason lies in the loss of conjugation between the C=O and C=C bonds after polymerization. In effect, the C=O bond strength increases, causing a reduction in the C=O peak intensity [18,126]. Due to the increased research activity towards developing new dimethacrylates that have no aromatic bisphenol A, the significance of this method has been growing in importance in recent years.

If a dimethacrylate does not provide a spectrum with a stable, well-resolved internal standard band, a calibration curve can be an option [101,127]. The calibration curve, representing a linear relationship between the absorption intensity and molar concentration of C=C bonds in a standard solution (moles C=C/mL), enables the determination of the C=C molar concentration in a monomer. By multiplying the C=C molarity by the methacrylate unit molecular weight (69.081 g/mol) and dividing the result by a monomer density (g/mL), a weight percentage of the methacrylate groups in monomer was obtained (*WPMG_mon_*). In order to use the calibration curve for the undiluted monomer, the experiment must be carried out using the same cell as the one used to build the calibration curve. The weight percentage of the methacrylate groups remaining in a polymer after polymerization (*WPMG_pol_*) was determined from a polymer IR spectrum. *WPMG_pol_* was calculated by dividing the C=C bond absorption intensity by the product of sample thickness and the optical constant (*K*) [101]. The *K* value of 0.64 was found by Rueggeberg [127]. Finally, *DC* was expressed according to the formula [101]:

(8) $$DC\left(\%\right)=\left[1-\frac{{\left(WPMG\right)}_{pol}}{{\left(WPMG\right)}_{mon}}\right]\times 100$$

Another option for the *DC* determination in dimethacrylate systems is the real-time infrared spectroscopy (RT FTIR). This method is based on a continuous measurement of the absorbance of the peak deriving from the vibration of C=C bonds during irradiation. Thanks to maintaining constant sample thickness, this method does not require the application of an internal standard or calibration curve. The *DC* determination is based on the observation of changes in the peak absorbance from the beginning of the experiment to its completion [5,29,31,54,83,91,106,108]:

(9) $$DC\left(\%\right)=\left[1-\frac{A_{t}}{A_{0}}\right]\times 100$$

where *A_t_* is the C=C bond absorbance at time *t*, *A*_0_ is the C=C bond absorbance before photoactivation (*t* = 0). 

Since this method is limited to samples polymerized during the FTIR experiment, it is especially valuable in studies on the photopolymerization kinetics [31,93].

#### 5.1.2. Raman Spectroscopy (RS)

Raman spectroscopy is a complementary vibrational technique for FTIR [112]. It offers the possibility of quantitative characterization of a polymerization extent in dimethacrylate systems, using similar peaks as FTIR. However, sample preparation for RS is much simpler (a sample does not require further processing) [84,112]. The internal standard method is also typically used for the determination of the *DC* with the use of RS. The *DC* is typically calculated by monitoring the percentage decrease in the intensity of the methacrylate double bond peak, at around 1640 cm^−1^, caused by polymerization, in respect to the aromatic peak at 1610 cm^−1^ [47,52,77,83,84,89,94,102,113,120,124,126,128,129,130]. The C-O-C ether stretching vibration band, occurring in the range of 1294 to 1118 cm^−1^ can be alternatively used [84,112]. Stretching and deformation vibrations of the dipole C–Cl and C–F bonds would be willingly used, occurring in the range of 800 to 200 cm^−1^, however, they rarely occur in a dimethacrylate structure [63]. NIR RS can also be utilized to monitor *DC* [128].

#### 5.1.3. Differential Scanning Calorimetry (DSC)

DSC is particularly well suited to studying the dimethacrylate photopolymerization kinetics [2,3,81,131,132,133]. The DSC apparatus has to be additionally equipped with a UV/VIS radiation source for such measurements. This technique, called differential photocalorimetry, working in an isothermal mode, is a convenient and easy way to determine the polymerization rate and degree of conversion of photoreactive systems.

Antonucci et al. determined *DC* by measuring the polymerization heat of dental dimetacrylates with the use of DSC working in dynamic mode. Polymerization was thermally activated with benzoyl peroxide [134].

Moszner et al. developed the procedure for the *DC* determination in real samples (taken from already existing material) using DSC [135]. The exothermal effect of the post-curing reaction was monitored in the dynamic mode. The *DC* was calculated by ratioing the post-polymerization heat and the heat of a total conversion according to the equation [135,136]:

(10) $$DC\left(\%\right)=\frac{\Delta H_{p}}{\Delta H_{100}}\times 100$$

where Δ*H*_100_ (J/mol) is the molar enthalpy of polymerization for the theoretical case of total conversion (*DC* = 100%, Δ*H*_100_ = 57.8 kJ/mol [137]), Δ*H_p_* (J/mol) is the molar enthalpy determined from the enthalpy value obtained in the DSC measurement (Δ*H_p,exp_* (J/g)), which was calculated according to the following equation:

(11) $$\Delta H_{p}\left(\frac{J}{\mathrm{mol}}\right)=\frac{\Delta H_{p,exp}\times MW}{f}$$

where *MW* is the monomer molecular weight (g/mol), *f* is the number of functional groups in the monomer (*f* = 2 in dimethacrylates).

#### 5.1.4. Solid State Nuclear Magnetic Resonance (ssNMR)

Solid-state NMR is another valuable technique for characterizing structural features of the polymers in the solid-state. In the works of Pereira et al. [116] and Morgan et al. [138], solid-state NMR has been shown to be a convenient method for measuring the degree of conversion in Bis-GMA containing polymers. ^13^C signals of carbonyl groups in the monomer and polymer methacrylate groups were observed at about 166 and 177 ppm, respectively. The integral values of these resonances were used to calculate the *DC*.

### 5.2. The Influence of Chemical Structure on the DC

The compilation of results from many studies leads to the following general conclusions. The *DC* notably depends on the monomer molecular elasticity and distance between methacrylate groups. The more elastic and longer the dimethacrylate molecule, the higher the *DC*. Dimethacrylates of stiff, spacious molecules often do not polymerize to the *DC* sufficiently enough to ensure material compliance with application requirements. Improved curing efficiency can be achieved by the addition of a certain amount of a reactive diluent [3,5,18,19,31,47,48,49,50,52,54,55,77,90,91,117,122,129,136]. High strength of intermolecular interactions between monomer molecules (hydrogen bonding) can also negatively affect the *DC* [3,18,31,50,77,78,117].

In the works of Gajewski et al. [19] and Sideridou et al. [18,117], the limiting *DC* in homopolymers of typical dental dimethacrylates was determined and explained. The homopolymers were arranged according to the following order by the increasing *DC*: Bis-GMA < UDMA < Bis-EMA < TEGDMA (Table 1). High molecular stiffness of Bis-GMA, resulting from the presence of aromatic rings and -OH groups, explains the lowest *DC* in the homopolymer. The limitations in rotational movement are so large that they prevent the Bis-GMA homopolymer from achieving the *DC* higher than 50%. Reversibly, the hydroxyl group lacking Bis-EMA was characterized by a higher possibility of rotational conformations, which resulted in a significant increase in *DC*. The combination of high elasticity and hydrogen bonding explains moderated *DC* of the UDMA homopolymer. The fully aliphatic character of TEGDMA and its shortest length was expressed in the highest *DC* [18,19,47,50,117]. In the study of Khatri et al. the Bis-GMA hydroxyl groups were blocked with the n-alkyl pendant substituents [66]. The *DC* in new polymers was higher than the *DC* in the Bis-GMA homopolymer. Additionally, it was found that the longer the substituent, the higher the *DC*. It shows, that due to tangled configuration that can be obtained by the aliphatic linear chains, their presence in the dimethacrylate does not limit curing quality. All these findings revealed a key role of hydrogen bonding in the evolution of a dimethacrylate polymer network. Hydrogen bonds can seriously restrict molecular mobility and diffusion-controlled termination, thus causing a reduction in the crosslinking efficiency. 

In research on copolymers, when Bis-GMA was combined with a more flexible comonomer, such as TEGDMA, an increase in *DC* was observed. The higher the TEGDMA content in the Bis-GMA/TEGMA copolymer, the higher the *DC* [5,54,55,70,91]. However, in the works of Elliott et al. [23] and Lopez-Suevos et al. [139], the risks associated with the increasing TEGDMA content were shown. The higher the TEGDMA concentration, the greater the tendency to form microgels, due to the greater likelihood of primary and secondary cyclization, which may jeopardize polymer packing density and lead to a looser network structure. It was confirmed in the work of Borges et al., where the influence of the Bis-GMA/TEGDMA ratio on the polymer network *DC* and crosslink density was determined [70]. The crosslink density increased as the TEGDMA content increased up to a limit of 50 wt% and then decreased, with a further increase of TEGDMA concentration. The *DC* in all polymers was similar and equaled around 95%. It informs that a certain number of double bonds were engaged in cyclization.

Test results for the *DC* in the Bis-GMA/Bis-EMA copolymers showed that *DC* increased as the Bis-EMA content increased [91], which is in agreement with the discussion presented above. The higher the possibility of rotational movement, the higher the *DC*.

A study of Barszczewska-Rybarek on a series of new urethane-dimethacrylates (Scheme 2) provided knowledge on the influence of the diisocyanate core chemical character and the oligoether chain length on the *DC* in homopolymers [90]. The *DC* in fully aliphatic polymers was found to be higher than the *DC* in polymers having aromatic and cycloaliphatic moieties. The presence of aromatic rings caused a greater decrease in *DC* than alicyclic structures. Monomers with asymmetrically substituted rings polymerized to higher *DC* when compared to those having symmetrically substituted rings. Additionally, the *DC* was closely related to the length of the oligooxyethylene chain, present in the monomer wings. The longer the chain, the higher the *DC*. However, monomers having tetraoxyethylene chains revealed a slight decrease in *DC*. The initial increase in *DC* was explained by the increased flexibility of monomer molecules. An increase in the possibilities of rotational conformations facilitated reaction-diffusion [2]. The final drop in *DC* was explained by a significant decrease in the concentration of double bonds, causing their remoteness. Park et al. studied *DC* in a series of copolymers of Bis-GMA with oligoethylene glycols dimethacrylates. The same as above, they found that the higher the number of oxyethylene units in a chain, the higher the *DC* [52].

Podgórski reported the results for a series of cured new dimethacrylates, synthesized from glycidyl methacrylate with dicarboxylic acid esters. The latter were obtained by the reaction of nadic anhydride with 1,3-propylene, 1,4-butylene, 1,5-penthylene and 1,6-hexylene glycols [119]. Chemical structures of new monomers were like Bis-GMA regarding the wings and differed regarding the cores. A very high *DC* was achieved by replacing the bisphenol A with the core, being the combination of alicyclic structures and oligomethylene chain. The longer the glycol, the higher the *DC*. The presence of the -OH groups did not negatively affect *DC*. The positive effect of the central aliphatic chain on *DC* was shown to be dominant. 

## 6. The Influence of Chemical Structure and Crosslink Density on Mechanical Properties

The decrease in crosslink density causes the deterioration of physicomechanical properties [5,32,41,47,53,55,56,63,85,92,94]. It comes from the decrease in *DC* and cyclization. The incomplete conversion results in the presence of pendant groups and chains ending with free methacrylate groups (Figure 1) [1,2,3,4,5,21]. *DC* does not reveal the presence of loops (Figure 1b). It was found that the longer the dimethacrylate monomer the greater the possibility of forming cycles [5,23,47,139]. For example, when comparing urethane-dimethacrylates with di- and tetraoxyethylene chains (Scheme 2), the latter one has a higher tendency to cyclization [31,48]. On the other hand, when comparing elastic TEGDMA to stiff and spacious Bis-GMA, the latter monomer has a lower tendency to cyclization [23,53,93,139]. Pendant chains and groups, as well as loops, are mechanically ineffective [140,141]. Pfeifer et al. revealed that they can act similarly to a plasticizer–by decreasing the network tightness they give rise to the increase in rotational movement [5]. 

Entanglements, another type of polymer network defect, work in the opposite way. As they represent additional restraints that magnify the change of entropy with deformation, their presence causes an increase in the physical crosslink density (Figure 1b) [20,21,22]).

The system composed of homo- and copolymers of Bis-GMA, Bis-EMA, UDMA, and TEGDMA (Scheme 1) was the object of many studies, which led to the general conclusions on the structure-property relationships [18,32,50,52,54,55,56,77,122,142,143]. The discussion presented below summarizes the results on the influence of chemical structure and degree of crosslinking on selected mechanical properties. The level of crosslinking was characterized by the *DC* (Table 1), the concentration of double bonds (Table 1) and the strength of hydrogen bonds (Table 4). The values of modulus of elasticity, mechanical strength, hardness and impact resistance of Bis-GMA, Bis-EMA, UDMA, and TEGDMA homopolymers are summarized in Table 5.

The Bis-GMA homopolymer network is characterized by the lowest crosslink density, which results from the lowest concentration of double bonds in the monomer and the lowest *DC* in the polymer (Table 1). Simultaneously, the Bis-GMA homopolymer is characterized by a high degree of physical crosslinking, due to the presence of strong OH-type hydrogen bonds. When homopolymers were compared by hardness, the Bis-GMA homopolymer was found to be the softest [32,142]. This indicates that hardness is very sensitive to the *DC* and chemical crosslink density. Higher values of the remaining mechanical properties suggest that the low level of chemical crosslinking can be compensated by physical crosslinking [32]. 

In the study of Gajewski et al., the flexural properties of Bis-EMA and Bis-GMA homopolymers were compared [19]. They found that poly(Bis-EMA), which is not physically crosslinked, was characterized by almost 100% higher *DC* and 20% higher flexural strength. The modulus of both polymers was similar. This indicates that the lack of hydrogen bonding can be partially compensated by the increase in *DC*. Flexural strength benefited more from the increase in *DC* than the modulus. In the study of Khatri et al., the influence of n-alkyl Bis-GMA substituent on the *DC* and flexural strength was shown [66]. By blocking the -OH groups, the flexural strength decreased. It was explained by the decrease in the chemical crosslink density (the *DC* increased, however, the concentration of double bonds decreased).

The TEGDMA homopolymer is fully aliphatic and has the highest crosslink density, resulting from the smallest monomer molecule and highest *DC* in the polymer (Table 1). It is not physically crosslinked. It was characterized by a relatively high modulus and the highest impact resistance among homopolymers. However, its flexural strength was the lowest [32]. This means that a high level of chemical crosslinking can ensure high modulus and impact resistance, however, it is insufficient to ensure high mechanical strength. 

The UDMA homopolymer is fully aliphatic and chemically as well as physically crosslinked. It was characterized by the highest flexural strength and hardness among homopolymers, which can be explained by high *DC* and hydrogen bonding. Additionally, this result indicates that the strength of physical crosslinks is not a key factor for these properties, as the comparison of the hydrogen bond strengths demonstrates. The strength of NH-type proton donor H-bond is 40% lower than that of the OH-type proton donor H-bond (Table 4). On the other hand, modulus and impact resistance of poly(UDMA) were the lowest, which leads to the conclusion that these properties of dimethacrylate polymer networks are especially sensitive to the strength of physical crosslinks [32,55].

The influence of chemical and physical crosslinking on tensile and flexural properties was also studied on Bis-GMA, UDMA and TEGDMA copolymers [3,5,18,32,55,56,93,102,122,142,143]. The increase in the TEGDMA content in Bis-GMA/TEGDMA copolymer resulted in an increase in tensile strength [56], but the decrease in flexural strength [56,122]. The decrease in flexural strength was explained by the decrease in physical crosslink density [18,32,93,122]. Tensile strength was found to be less sensitive to physical crosslinking and more sensitive to chemical crosslinking, which increased with the TEGDMA incorporation. The addition of UDMA into copolymers resulted in an increase in both tensile and flexural strength [102,122]. It confirmed that in the case of flexural strength, the presence of physical crosslinks is more important than their strength [32,122].

The results for modulus of Bis-GMA, UDMA, TEGDMA copolymers (filled with silanized glass) were reported in the study of Asmussen et al. [56]. The modulus of Bis-GMA/TEGDMA copolymer increased by adding a certain TEGDMA content, which was 40 wt% (the positive effect of the increasing *DC* and crosslink density). Higher TEGDMA contents had a negative effect on modulus and caused its reduction. It was explained by the decreasing physical crosslink density and concentration of stiff aromatic structures. The same trend was observed when UDMA was added. A maximum in modulus of elasticity was achieved at 10 wt% of the UDMA content. 

In the work of Sideridou et al., the effect of copolymer composition on Young’s modulus was determined [53]. It was found, that Bis-GMA/TEGDMA copolymers had significantly higher values for Young’s modulus than those predicted by the linear dependence of the values on the copolymer composition. On the contrary, the copolymers of Bis-GMA/UDMA and Bis-GMA/Bis-EMA had Young’s modulus values slightly lower than those predicted. It was explained by lower *DC* in the TEGDMA-free copolymers and the presence of spacious pendant groups, constructed of long, massive Bis-GMA, Bis-EMA, and UDMA molecules, which affected a polymer structure by plasticizing it [5,53]. 

Hardness is another important mechanical property of poly(dimethacrylate)s. It was found that the hardness of the Bis-GMA homopolymer was lower by around 20% than the Bis-GMA/TEGDMA copolymer [32]. In the other study, copolymers of Bis-GMA with increasing TEGDMA content were characterized. It was found that the hardness of BisGMA/TEGDMA copolymers was unaffected by the TEGDMA content [143]. The results for the *DC* in the Bis-GMA homo- and copolymers, collected by Collares et al., can help to clear up that discrepancy [91]. The *DC* in poly(Bis-GMA) can reach the maximum of 40% [18,47,143]. The incorporation of TEGDMA in the amount of 10 wt% caused the *DC* increase of up to 60%. Further increase in the TEGDMA content resulted in further *DC* increases. The *DC* of 76% was found for the Bis-GMA/TEGDMA 10/90 wt% copolymer. It suggests that hardness becomes less sensitive to *DC* as it exceeds 50%. Copolymerization of Bis-GMA and TEGDMA with UDMA resulted in an additional increase in hardness [32]. This tendency was also noticed when other urethane-dimethacrylate monomers (Scheme 2) were used in copolymerizations [55]. This effect was explained by the synergistic effect of a high degree of double bond conversion and hydrogen bonding. 

By comparing the impact resistance of Bis-GMA/TEGDMA and Bis-GMA/TEGDMA/UDMA copolymers it should be noted that, as the UDMA was added, its value decreased, despite the increase in the *DC* [32]. The moderate strength of hydrogen bonds was pointed at again to explain this result. In the study of Pfeifer et al., the influence of Bis-EMA on the Bis-GMA/Bis-GMA copolymer impact resistance was determined [102]. It was found that impact resistance decreased as Bis-EMA was added. It was also explained by the decrease in the overall strength of intermolecular interactions. As the -OH group concentration decreased, the number of possible sites of physical crosslinking was reduced [102,115]. 

The detailed analysis of polymer networks of diacrylate analogous of dental dimethacrylates showed that the higher the crosslink density the higher the impact resistance [45]. As *q* results from the *DC*, the limit of 70% of the latter was found, below which intermolecular forces start to play a key role in impact resistance. The higher the strength of hydrogen bonds and the higher their concentration, the higher the impact resistance. The influence of crosslink density on the failure behavior in crosslinked polymers was confirmed by molecular dynamics simulations [72].

The detailed study on a series of Bis-GMA/TEGDMA AgNP nanocomposites [92] and urethane-dimethacrylate homopolymers [35,48] confirmed the conclusion that the incomplete conversion adversely affects mechanical properties. Additionally, results of the studies on AgNP nanocomposites allowed for constructing the following order of properties by increasing sensitivity to the *DC*: hardness < modulus < bending strength < impact resistance. Increasing AgNP concentration in nanocomposites usually caused decreases in these properties, which coincided with the decreasing *DC*. The detailed analysis of variations in mechanical properties as a function of the *DC* of thermally cured samples established that hardness increased throughout the polymer series, regardless of the decrease in *DC*. Remaining mechanical properties increased at lower AgNP concentrations, and then decreased. Modulus only decreased when *DC* dropped below 50%. Bending strength decreased when *DC* was down to 70%. Impact resistance was the most sensitive to *DC* and decreased when *DC* was down to 77% [92]. 

As can be seen, the impact strength of dimethacrylate polymers is a complex phenomenon. It should have high values in order to withstand forces generated during chewing and to prevent accidental fracture [144,145]. However, they are usually lower than expected [26]. Impact strength depends on many physicochemical factors, such as the monomer molecule elasticity, chemical crosslink density, physical crosslinking, degree of conversion and morphology. The higher the chain elasticity [35], crosslink density [32], and the strength of hydrogen bonds [32], the higher the impact resistance. The lower the *DC*, the lower the impact resistance [32,45,92]. The lower the DC, the greater the role of morphological heterogeneity, resulting from microgel formation [45], which is described in the following section. Additionally, impact strength can be radically reduced due to the presence of air bubbles trapped in the material [92].

## 7. The Morphology

Generally, poly(dimethacrylate)s are regarded as non-crystalline, amorphous, highly crosslinked polymers. Their supramolecular structure consists of microgel agglomerates [1,2,3,4,5,23,24,25,26,27]. Since it is only a short-range order of supramolecular organization, its characterization is complicated. Many works confirmed the formation of distinct nodular morphology of poly(di(meth)acrylate)s. It was visualized through microscopic observations with the use of atomic force microscopy (AFM) [26,32,33,34,36,38,71,123,136,146,147,148] and scanning electron microscopy (SEM) [55,123]. It is only in recent years that methodologies for quantitatively characterizing the morphology of dimethacrylate polymer networks have been developed. They utilize AFM supported with advanced mathematical tools [33,34,38,148], X-ray powder diffractometry (XRPD) [32,35,45,136] as well as techniques of thermal analysis: dynamic-mechanical analysis (DMA) [1,42,52,69,70,71,149,150,151,152,153], differential scanning calorimetry (DSC) [39] and thermogravimetry (TG) [154,155,156].

### 7.1. Qualitative Microscopic Characterization 

The AFM images of poly(dimethacrylate) fractures revealed that their morphology consists of globular domains with diameters in the range of 10–100 nm [26,32,33,34,36,38,71,123,136,146,147,148]. They were attributed to highly crosslinked microgel agglomerates embedded in a less crosslinked matrix. The qualitative (agglomerate shape) and quantitative (agglomerate dimensions) differences were rarely observed between dimethacrylate polymers. Exceptionally, the Bis-GMA homopolymer revealed the existence of morphology presenting certain specific features. In contrast to the conventional spherical agglomerates, the morphology of poly(Bis-GMA) consisted of elongated “rods”, having a diameter of approximately 70 nm and length of up to 500 nm [32]. This phenomenon coincided well with the results from SEM observations. The images of Bis-GMA homopolymer and its copolymer with 20 wt% of TEGDMA revealed a distinctive morphology, which consisted of long, needle-shaped, morphological objects. The remaining dimethacrylate homo- and copolymers showed a more uniform, smooth morphology [55]. 

Additionally, SEM and AFM observations led to the conclusion that the irradiation direction determines the cross-section morphology. The fracture surfaces perpendicular to the irradiation direction revealed the presence of parallel lines. The anisotropy in the polymerization shrinkage, caused by the high diameter to thickness ratio of mold was identified as the most likely reason for this phenomenon [32,55].

### 7.2. Quantitative AFM Characterization 

#### 7.2.1. Phase Imagining 

The AFM morphology was quantified by using the phase imaging AFM of local viscoelasticity of the Bis-GMA and Bis-EMA acrylate analogue polymers [37,38,39]. The results provided valuable information about the kinetics of diacrylate radical polymerization, which can be transformed into dimethacrylates. The formation of three types of domains, with a varied elastic response, corresponding to varied crosslink density, was detected. However, any quantitative differences in morphologies depending on the chemical composition nor the irradiation type were observed. 

#### 7.2.2. Roughness Analysis 

The globular AFM topography can be characterized using roughness analysis. There are several parameters available with common image processing software packages. They include the density of summits, mean summit curvature, surface area ratio, root-mean-square roughness. In the work of Munz, these parameters were readily used as descriptors for spatial resolution of diacrylate hydrogel polymer networks, consisting of globular domains with diameters in the range of ~10–100 nm [148]. In dental material studies roughness analysis is typically used to characterize composite surface morphology, depending on the filler type and polishing technique [147,157,158]. In the work of Lungu et al. this procedure was used for the characterization of the UDMA-silsesquioxane nanocomposites [123]. It was found that the addition of silsesquioxane caused a decrease in matrix transparency and it was attributed to the formation of agglomerates. 

#### 7.2.3. Fractal Analysis

Fractal analysis of the AFM fracture surfaces of poly(dimethacrylate)s was successfully applied for the quantitative description of their morphology [33]. For that purpose: the fractal dimension (*D_F_*) and its extension–the generalized fractal dimension (*D_q_*) were determined. From the difference between the extreme values of *D_q_*: D_−∞_ and D_+∞_, the Δ*D* parameter was calculated. Its value refers to the degree of surface differentiation and tends towards zero with an increasing degree of homogeneity [159]. Fracture topography was further characterized by analyzing their profile line, which was characterized by the modified fractal dimension (*D_β_*). As a result, several linear relationships between fractal morphology, chemical structure and selected physicomechanical properties of poly(dimethacrylate)s obtained by homo- and copolymerizations of Bis-GMA, UDMA, and TEGDMA were found. The higher the *D_F_* and *D_β_*, the higher the hardness. The higher the *D_F_*, the higher the density. A linear relationship between Δ*D* and impact resistance was also found. The higher the Δ*D*, i.e., the greater the polymer heterogeneity, the lower the impact strength. That finding indicated that this mechanical property is governed by the degree of self-similarity. 

The detailed analysis of the relationships between the chemical structure and fractal parameters led to the following conclusions [159,160]. The TEGDMA homopolymer morphology was characterized by the highest *D_F_* as well as *D_β_* and the lowest Δ*D*, indicating the highest structural homogeneity of this polymer network. It was explained by the complex (tangled) conformations, which linear aliphatic chains can adopt [66] and decreased free volume, due to the increased degree of conversion [5], both enabling the highest degree of space fitting. In opposite, the poly(Bis-GMA) fracture was characterized by the lowest *D_F_* and *D_β_* , which corresponds to the lowest degree of space fitting, due to high Bis-GMA rigidity. The results for Δ*D* lead to an explanation of the lowest impact resistance of the UDMA homopolymer. Δ*D* of poly(UDMA) was the highest. A lower Δ*D* of Bis-GMA homopolymer network indicated that its structure can be considered as more homogeneous (tighter), due to a higher strength of physical crosslinks in poly (Bis-GMA) than in poly(UDMA) (Table 4). Copolymerizations resulted in an increase in the fractal dimensions. Thus, the conclusion that TEGDMA plays a crucial role in the development of the dimethacrylate polymer network morphology by reducing its heterogeneity was drawn. 

#### 7.2.4. Percolation Theory

Another study on the model system of dental dimethacrylate homo- and copolymers showed that their AFM fracture surfaces have a percolating structure. For this purpose, the percolation probability (*P*) and length of the percolation path (*L*) were determined and their values were related to the selected properties of studied polymer networks [34]. Analysis of the results indicated that both parameters, *P* and *L*, are strongly dependent on the presence of the physical crosslinks, formed by hydrogen bonds. Their strength and the degree of conversion play a further role. However, it was noticed that the higher the crosslink density and hydrogen bond strength, the higher the percolation probability, i.e., the larger clusters are formed during polymerization. 

The impact strength of the UDMA homopolymer was a special subject of interest due to its higher than expected brittleness. Studies on the quantitative characterization of the AFM fracture surfaces with the use of the percolation theory tools clearly showed that the impact strength increases with increasing percolation probability. Also, poly(UDMA) was characterized by the lowest *P*.

Additionally, the beneficial role of TEGDMA as a comonomer in the formation of a dimethacrylate polymer network structure was reconfirmed. Its presence allows for the formation of large percolating clusters and long percolation paths, consisting of chemical and physical crosslinks, with the aid of ester and ether proton acceptors.

### 7.3. Quantitative XRPD Characterization 

XRPD can also be recognized as a valuable tool for the quantitative characterization of supramolecular organization in dimethacrylate polymer networks. 

In several studies, the average dimensions of microgel agglomerates (*D*) were calculated by using the peak half-width and the Scherrer equation [32,35,45,136]. Their values were of a few nanometers, which confirmed that dimethacrylate polymer networks have a short-range molecular order [161]. Nevertheless, the *D* values were varied and depended on the polymer chemical structure and increased according to the following order: TEGDMA < Bis-GMA/TEGDMA < UDMA < Bis-GMA < Bis-GMA/TEGDMA/UDMA.

These studies revealed that the morphology of dimethacrylate polymer networks is influenced by the monomer molecule dimensions, packing abilities and strength of physical crosslinks. The more spacious the monomer molecule and the higher the hydrogen bond strength, the higher *D*. The more compact packing (higher elasticity, resulting in higher rotational possibilities), the lower *D*.

In the work of Barszczewska-Rybarek, it was found that the presence of massive agglomerates can negatively affect the impact resistance of poly(diacrylate)s. The ability to form the strongest hydrogen bonds in the Bis-GA/TTEGDA/UDA copolymer (the acrylate analogous to Bis-GMA/TEGDMA/UDMA) favored the formation of massive clusters. However, their concentration within the less crosslinked matrix was insufficient to effectively withstand the energy of the impact [45].

### 7.4. Thermal Analysis 

Recently, the quantitative characterization of the structural heterogeneity of dimethacrylate polymer networks was extended to the use of thermal analysis techniques: dynamic-mechanical analysis (DMA) [1,42,52,69,70,71,149,150,151,152,153], differential scanning calorimetry (DSC) [39] and thermogravimetry (TG) [154,155,156]. The bimodal character of the peaks present on the thermograms in the glass transition region or the decomposition region, indicates the presence of two phases significantly varying in crosslink density. The temperature range in which the physical change or chemical reaction occurs must be determined. The narrower the temperature range the more homogeneous the network. In the case of DMA, the changes in the dynamic storage modulus and the loss tangent are observed, which occurs during the glass transition. Heterogeneity was quantified by the determination of the peak width at half-height [1,42,52,69,70,71,149,150,151,152,153]. Additionally, the network heterogeneity can be assessed by the storage modulus decrease in the glass transition region. It was found that, the more heterogeneous the network, the more rapid decrease in the storage modulus in the region of low frequencies. It was also found that the loss modulus in the region of its maximum is very slightly sensitive to the “long-range” network heterogeneity [149]. In the TG studies, the gradual weight loss at the polymer degradation temperature is determined [154,155,156]. In the DSC studies, the changes in the heat capacity during glass transition as well as the enthalpy of curing are evaluated [39]. However, this information does not provide answers to questions about the size and arrangement of morphological objects.

The literature provides a detailed DMA study on the influence of the Bis-GMA/TEGMA ratio on the polymer network heterogeneity, which was characterized by the crosslink density (calculated from *E’*) and tan delta peak width [70]. The latter decreased as the TEGDMA content increased up to a certain limit and then increased, with a further increase of TEGDMA concentration. The Bis-GMA/TEGDMA 60/40 copolymer was characterized by the lowest tan delta peak width. i.e., it was recognized as the most homogeneous. That result was explained by dense packing and uniform free volume distribution [70]. The Bis-GMA/TEGDMA 20/80 copolymer was characterized by the lowest crosslink density and the broadest tan delta peak. That result, in relation to a very high *DC* (around 95%), indicated the greatest heterogeneity and the high tendency to cyclization represented by TEGDMA [70,152].

## 8. Biocompatibility of Dental Dimethacrylate Polymer Networks

The biocompatibility of dental materials is an important issue. It is closely connected with the polymer network structure development. In general, this term describes the material ability to be in contact with a living system without producing adverse effects by not being toxic, injurious, physiologically reactive and not causing immunological rejection [162]. In fact, it is a complex phenomenon, which involves biological interactions, patient risks, clinical experience, and engineering expertise. For that reason, no single test is required to evaluate the biological efficacy of material [163]. There is no specific procedure for characterizing the biocompatibility of dental materials. Several researchers recommend using standardized testing procedures for the evaluation of the biocompatibility of medical devices [163,164]. Those documents include ANSI/ADA standard No. 41 [165] and the ISO 10993 series of standards [166]. 

Dimethacrylate polymer networks are generally non-toxic [9]. Their toxicity results from leaching of low molecular weight substances, which can have a harmful effect on oral tissues [167,168,169]. Low molecular substances can come from the incomplete conversion and chemical degradation of dimethacrylate polymer network [168,169]. Several adverse reactions were reported after the application of dimethacrylate dental material. These include migraine, amenorrhea, fatigue, sinusitis, insomnia etc. The reliability of those complaints is difficult to verify, especially since several researchers pointed out that they can have a psychosomatic background [169].

Unreacted dimethacrylate monomer can be eluted by saliva [169]. Water and ethanol are the most popular solvents, occurring in the oral environment [53,58,102]. The local concentrations of leached monomers can be in the millimolar range, which is high enough to induce a variety of adverse biological effects [167]. It was found that the higher the degree of conversion, the lower the possibility of unreacted monomer content in the dimethacrylate matrix [170]. It was also found that the presence of unpolymerized surface layer, resulted from the oxygen inhibition, is a significant source of the unpolymerized monomer and increases composite cytotoxicity [169]. For this reason, mechanical removal of the oxygen inhibited layer could be recommended [171]. 

Typical dental dimethacrylates were thoroughly examined for cytotoxic properties. Issa et al. [172] and Reichl et al. [173] tested the cytotoxicity of dental dimetacrylates by using the MMT (3-(4,5-dimethylthiazol-2-yl)-2,5-diphenyltetrazolium bromide) and LDH (lactate dehydrogenase) assays. The TC_50_ values (concentrations altering MTT and LDH activity by 50%) were similar in both assays and ranged from around 0.32 to 6.7 mM. Monomers were arranged by increasing toxicity (increasing TC_50_) according to the following order: HEMA (2-hydroxyethyl methacrylate) < DMAEMA (2-(dimethylamino)ethyl methacrylate, photoinitiation accelerator) < TEGDMA < UDMA < Bis-GMA [172]. Dental dimethacrylate monomers were also tested for acute toxicity. It was found that LD_50_ (the median lethal dose of a toxin, which corresponds to the dose required to kill half the members of a tested population) of Bis-GMA and UDMA was of > 5000 mg/kg body weight (rats), whereas LD_50_ of TEGDMA equaled to 10,837 mg/kg body weight (rats). As all the LD_50_ values were greater than 2000 mg/kg body weight, it means that these monomers cannot be classified as toxic [169]. The ED_50_ (the dose that produces a biological response in 50% of the population that takes it) for dental dimetnacrylates was also determined. According to this parameter, dimethacrylates can be arranged according to the following order: Bis-GMA (0.08–0.14 mM) < TEGDMA (0.12–0.26 mM) < UDMA (0.06–0.47 mM) < Bis-EMA (0.21–0.78 mM) < HEMA (1.77–2.52 mM) [174]. A summary of these results leads to the conclusion that TEGDMA is less toxic than Bis-GMA and UDMA. They are also in line with the general principle that dimetacrylates are more toxic than monometacrylates [169,175]. 

The second reason of the presence of leachable components in a composite dental material is hydrolysis or enzymatic degradation of a dimethacrylate polymer network [168,169,175]. Several products of Bis-GMA and Bis-EMA biodegradation, such as bisphenol A, bisphenol A diglycidylether and bisphenol A dimethacrylate were found to have estrogenic-like effects [168,169,175,176,177]. It was also found that Bis-EMA undergoes biodegradation to a lesser degree than Bis-GMA [169]. However, some researchers indicate that use of Bis-GMA based dental materials do not cause a clinically relevant estrogen-like effect on patients. Thus, the postulated estrogen-like effect is no reason to restrict indications for these dimethacrylate-based materials [169]. 

## 9. Conclusions

Material characterization and design are essential to better understanding its physicochemical and mechanical properties. In particular, the determination of relationships between structure and properties at different scales can lead to the improvement of material performance. This review is well-suited to the contemporary trends in the detailed characterization of polymeric materials, which are focused on the development of modern research methodologies and advanced interpretation of their properties. It can be expected that the issues presented in this review would be beneficial for the engineering of dimethacrylate dental materials.

It can be concluded that the higher the *DC* and the more homogeneous the morphology of a dimethacrylate polymer network, the better its mechanical performance. Molecular elasticity, resulting from higher possibilities of rotational movement and compact packing, usually positively affects the *DC* and morphology. Morphological heterogeneity arises from the formation of microgel agglomerates. The bigger the monomer molecule and the stronger the hydrogen bonds, the higher the network heterogeneity. On the other hand, hydrogen bonding positively affects many mechanical properties, especially impact resistance. Strong hydrogen bonds present in the Bis-GMA structure, by acting as physical crosslinks, are capable of compensating for the negative effect of insufficient *DC* on mechanical properties, especially impact resistance. The strength of H-bonds formed by the UDMA imine proton is insufficient to ensure high impact resistance. On the other hand, it is enough to ensure high hardness. 

Copolymerization of basic monomers, such as Bis-GMA and UDMA, with reactive diluents, such as TEGDMA, having small and flexible molecules, usually causes an increase in the polymer network homogeneity and mechanical performance. 40 wt% of TEGDMA was found as the optimum content in a copolymer. Its higher amount can cause an increase in the morphological heterogeneity due to cyclization.

## Figures and Tables

**Table 1:** Properties of popular dimethacrylate monomers and the degree of conversion in the corresponding homopolymers.

Monomer	Molecular Weight (g/mol)	Concentration of Double Bonds (mol/kg)	Viscosity (Pa∙s)	Degree of Conversion (%)
Bis-GMA	511	3.90	1200 ^1^	39.0 ^1^/34.5 ^2^
Bis-EMA (n+m = 4)	540	3.70	0.9 ^2^	75.5 ^2^
UDMA	470	4.25	23.1 ^1^	69.6 ^1^/72.4 ^2^
TEGDMA	286	6.99	0.011 ^1^	75.5 ^1^/82.5 ^2^

^1^ Taken from [18]; ^2^ Taken from [19].

**Table 2:** Water sorption of typical dental dimethacrylate homopolymers.

Monomer	Water Sorption (µg/mm^3^)
Bis-GMA	32.18 ^1^, 33.49 ^2^
Bis-EMA (n = 4)	20.10 ^2^
UDMA	23.85 ^1^, 29.46 ^2^
TEGDMA	66.93 ^1^, 69.51 ^2^

^1^ As cited in [48]; ^2^ As cited in [53].

**Table 3:** The influence of hydrogen bonding on the location of absorption bands of the N–H and C=O groups in polyurethane systems, represented in Figure 2b.

**Proton Donor Group**	**H-Bond Type**	**Wavenumber (cm^−1^)**
ν(N–H)	–	3445–3450 ^1^
ν(N–H)	N–H...N–H	3315–3340 ^1^
ν(N–H)	N–H...O	3260–3290 ^1^
**Proton Acceptor Group**	**H-Bond Type**	**Wavenumber (cm^−1^)**
ν(C=O)	–	1730–1740 ^1^ 1721–1726 ^2^
ν(C=O)	C=O...H–N	1703–1710 ^1^
ν(C=O)	C=O...H–O	1712–1719 ^2^ 1705 ^2,3^

^1^ As cited in [80]; ^2^ As cited in [81]; ^3^ deconvoluted H-bonding.

**Table 4:** Types of hydrogen bonds and corresponding energies (adapted from [32]).

The Hydrogen Bond Type	Energy (kJ/mol)
Ο−Η.......Ν	29
Ο−Η.......Ο	21
Ν−Η.......Ν	13
Ν−Η.......Ο	8

**Table 5:** Mechanical properties of popular dimethacrylate homopolymers.

Monomer	Young’s Modulus (MPa)	Flexural Modulus (MPa)	Flexural Strength (MPa)	Hardness (MPa)	Impact Resistance (kJ/m^2^)
Bis-GMA	1427 ^1^	1000 ^2^	72.4 ^2^	73 ^3^	6.41 ^4^
Bis-EMA (n = 4)	744 ^1^	1100 ^2^	87.3 ^2^	–	–
UDMA	1405 ^1^	1800 ^2^	133.8 ^2^	162 ^3^	4.85 ^4^
TEGDMA	1134 ^1^	1700 ^2^	99.1 ^2^	129 ^3^	8.83 ^4^

^1^ Taken from [53]; ^2^ Taken from [19]; ^3^ Taken from [48]; ^4^ Taken from [35].
